# Revealing the three-dimensional murine brain microstructure by contrast-enhanced computed tomography

**DOI:** 10.3389/fnins.2023.1141615

**Published:** 2023-03-23

**Authors:** Tim Balcaen, Catherine Piens, Ariane Mwema, Matthieu Chourrout, Laurens Vandebroek, Anne Des Rieux, Fabien Chauveau, Wim M. De Borggraeve, Delia Hoffmann, Greet Kerckhofs

**Affiliations:** ^1^MolDesignS, Sustainable Chemistry for Metals and Molecules, Department of Chemistry, KU Leuven, Leuven, Belgium; ^2^ContrasT Team, Institute of Mechanics, Materials and Civil Engineering, Mechatronic, Electrical Energy and Dynamic Systems, UCLouvain, Louvain-la-Neuve, Belgium; ^3^Pole of Morphology, Institute of Experimental and Clinical Research, UCLouvain, Brussels, Belgium; ^4^Advanced Drug Delivery and Biomaterials, UCLouvain, Brussels, Belgium; ^5^Bioanalysis and Pharmacology of Bioactive Lipids, UCLouvain, Brussels, Belgium; ^6^Université Claude Bernard Lyon 1, Centre National de la Recherche Scientifique, Institut National de la Santé et de la Recherche Médicale, Centre de Recherche en Neurosciences de Lyon U1028 UMR 5292, Bron, France; ^7^Laboratory of Biomolecular Modelling and Design (LBMD), Biochemistry, Molecular and Structural Biology, Department of Chemistry, KU Leuven, Leuven, Belgium; ^8^Prometheus, Division of Skeletal Tissue Engineering, KU Leuven, Leuven, Belgium; ^9^Skeletal Biology and Engineering Research Center, KU Leuven, Leuven, Belgium; ^10^Department Materials Engineering, KU Leuven, Leuven, Belgium

**Keywords:** contrast-enhanced computed tomography, cuprizone demyelination, contrast-enhancing staining agents, 3D virtual histopathology, polyoxometalates, organic iodinated contrast-enhancing staining agents

## Abstract

To improve our understanding of the brain microstructure, high-resolution 3D imaging is used to complement classical 2D histological assessment techniques. X-ray computed tomography allows high-resolution 3D imaging, but requires methods for enhancing contrast of soft tissues. Applying contrast-enhancing staining agents (CESAs) ameliorates the X-ray attenuating properties of soft tissue constituents and is referred to as contrast-enhanced computed tomography (CECT). Despite the large number of chemical compounds that have successfully been applied as CESAs for imaging brain, they are often toxic for the researcher, destructive for the tissue and without proper characterization of affinity mechanisms. We evaluated two sets of chemically related CESAs (organic, iodinated: Hexabrix and CA4+ and inorganic polyoxometalates: 1:2 hafnium-substituted Wells-Dawson phosphotungstate and Preyssler anion), for CECT imaging of healthy murine hemispheres. We then selected the CESA (Hexabrix) that provided the highest contrast between gray and white matter and applied it to a cuprizone-induced demyelination model. Differences in the penetration rate, effect on tissue integrity and affinity for tissue constituents have been observed for the evaluated CESAs. Cuprizone-induced demyelination could be visualized and quantified after Hexabrix staining. Four new non-toxic and non-destructive CESAs to the field of brain CECT imaging were introduced. The added value of CECT was shown by successfully applying it to a cuprizone-induced demyelination model. This research will prove to be crucial for further development of CESAs for ex vivo brain CECT and 3D histopathology.

## 1. Introduction

The brain is a complex organ that enables organisms to think, remember, feel and move (Wang et al., 2020). Despite the scientific community’s interest in this organ, many unknowns remain, among others, about the structure-function relationship that allows the brain to perform its daily tasks (Batista-Garcia-Ramo and Fernandez-Verdecia, 2018). This relationship assumes that the anatomical connectivity can partially explain the functional connectivity of the brain. However, more in-depth knowledge on the brain microstructure is required to verify the structure-function relationship. The current gold standard for structural characterization of biological tissues, both in research and in the clinical setting, is classical two-dimensional (2D) histological assessment. The workflow of this methodology consists of embedding a tissue sample in a matrix, which is then sectioned into 2D slices. These sections are analyzed, most often using optical microscopy. However, the refractive index of the tissue often resembles the refractive index of the embedding matrix. Therefore, chemical stains and dyes are used, which specifically interact with tissue constituents, cell types and biomolecules, allowing the identification of morphological and biochemical structures (Horobin, 1975; Longo et al., 2012; Veuthey et al., 2014). Although this technique has a high discriminative power in discerning (sub)cellular structures, it is destructive for the tissue and does not provide a full 3D image. Moreover, embedding and cutting the tissue is time-consuming and is prone to cause deformations (e.g., by dehydration-induced shrinkage) of the tissue and cell structure (McInnes, 2005). Advances in high-resolution three-dimensional (3D) *ex* vivo imaging techniques may enable researchers to visualize the microstructure of the brain more accurately and, as a result, aid in unraveling the structure-function relationship.

Although multiple 3D neuroimaging methods exist, currently three non-destructive imaging techniques that enable researchers to study the complete 3D microstructure of tissues are receiving increased attention: light-sheet microscopy (LSM), micro magnetic resonance (microMRI) imaging, and X-ray microfocus computed tomography (microCT) imaging. All three techniques allow obtaining information complementary to each other and to 2D optical microscopy. LSM generates 3D images of a specimen by optical sectioning and can be applied both *in vivo* for functional imaging and *ex vivo* for structural imaging. The *ex vivo* approach however often requires clearing and expansion of the tissue sample (Hillman et al., 2019). LSM reaches up to single-cell resolution and can provide (bio)chemical information, but is limited in the size of specimens that can be imaged (Ahrens et al., 2013; Taranda and Turcan, 2021; Perens and Hecksher-Sorensen, 2022). MicroMRI imaging allows to study the brain both *in vivo* and *ex vivo* (Natt et al., 2002; Boretius et al., 2009; Chuang et al., 2011). This technique uses the tissue-specific magnetic properties of hydrogen atoms to generate 3D images with or without the use of contrast agents (Ishiguchi and Takahashi, 2010; Paschke et al., 2018). Even though microMRI does not need ionizing radiation, has an excellent discriminative power and offers both structural and chemical information, it is time-consuming, expensive, requires extensive knowledge of the technique and is unable to achieve the spatial resolution of microCT (e.g., microMRI achieves 20 μm voxel size after 24 h of scanning) (Chen et al., 2018). MicroCT-based imaging methods exploit the interaction of X-rays with tissue constituents to study the 3D microstructure of tissues *ex vivo*. In comparison to microMRI, this technique achieves higher spatial resolution in shorter timeframes, is less expensive and requires less extensive technical knowledge. However, to obtain a high discriminative power, methods for enhancing the contrast between different tissue constituents are required. At present, the most promising contrast-enhancing methods include phase-contrast microCT (PCCT) (Takeda et al., 2013; Barbone et al., 2021; Rodgers et al., 2021, 2022; Chourrout et al., 2022a,b; Palermo et al., 2022) and contrast-enhanced microCT (CECT) (Rodrigues et al., 2021). While PCCT makes use of the refractive properties of the X-rays, CECT aims to increase the X-ray attenuating properties of different tissue constituents (e.g., white matter versus gray matter) by incubating or infiltrating the tissue with contrast-enhancing staining agents (CESAs). Despite the fact that PCCT is able to generate high-quality images of weakly attenuating materials without the use of CESAs, this method in most cases requires the use of synchrotron facilities, in which a coherent X-ray beam can be generated. In combination with the complex reconstruction algorithms necessary to obtain the 3D datasets and the limited sample volume, this renders PCCT less applicable for everyday use. CECT in comparison does not require coherent X-ray beams, allowing data acquisition with less specialized hardware. Hence, CECT has been receiving increased attention and as a result has been widely investigated for imaging the nervous system of a variety of animal species using various CESAs (Supplementary Table 1).

According to existing literature, mainly inorganic CESAs have been explored for CECT imaging of brain tissue. These include simple, but rather toxic and invasive compounds (i.e., osmium tetroxide (OsO_4_) (Ribi et al., 2008; White and Brown, 2015; Parlanti et al., 2017; Chen et al., 2018; Masis et al., 2018; Pinto et al., 2022), potassium dichromate (K_2_Cr_2_O_7_) (Herrera et al., 2018), uranyl acetate (([UO_2_(CH_3_CO_2_)_2_H_2_O)]H_2_O)_n_) (Smith et al., 2016; Parlanti et al., 2017), silver nitrate (AgNO_3_) (Mizutani et al., 2010), phosphotungstic acid (PTA) (Buytaert et al., 2014; Smith et al., 2016; Zikmund et al., 2018; Rother et al., 2021) and alcoholic (Buytaert et al., 2014; Smith et al., 2016; Zikmund et al., 2018; Kavkova et al., 2021; Pinto et al., 2022) or aqueous (Buytaert et al., 2014; Anderson and Maga, 2015; Choi et al., 2017; Heimel et al., 2019; Lombardi et al., 2019; Llambrich et al., 2020; Feldman et al., 2022) iodine-based formulations). Furthermore, staining with Lugol’s iodine solution, alcoholic iodine solutions and PTA resulted in substantial tissue shrinkage and hence possible tissue deformation, which prevent correct and quantitative characterization of the tissue microstructure (Vickerton et al., 2013; Wong et al., 2013; Buytaert et al., 2014; Balint et al., 2016; Koc et al., 2019; Dawood et al., 2022). Multiple iodinated organic molecules have also been applied as CESAs, such as Hypaque^®^-76 (de Crespigny et al., 2008) and Iopamiron^®^ (Saito and Murase, 2012; Udagawa et al., 2019). In many of the aforementioned cases where CESAs have been screened, the link between the chemical properties of the CESA, the solvent (e.g., aqueous buffer solution) and the observed affinity (i.e., the staining mechanisms) towards the different brain tissue constituents has not been thoroughly investigated, notwithstanding the fact that some studies do give well-substantiated hypotheses (Saito and Murase, 2012, 2013; Herrera et al., 2018).

To address the needs for non-destructive, yet effective CESAs with a more fundamental understanding of their affinity towards different brain constituents, we screened two sets of chemically related compounds for their staining potential in CECT of murine hemispheres: two inorganic polyoxometalate CESAs (1:2 Hafnium-substituted Wells-Dawson polyoxometalate (Hf-WD 1:2 POM) and the Preyssler anion) and two organic CESAs (Hexabrix^®^ and CA4+). These compounds, except Preyssler anion, have already been successfully explored as CESAs for CECT on various tissue types other than nervous tissue (Kerckhofs et al., 2014, 2018; Karhula et al., 2017; Stewart et al., 2017; de Bournonville et al., 2020; Baer et al., 2021). We studied their diffusional properties into the organ, their specificity towards certain tissue constituents and their potential for obtaining quantitative 3D structural information. Additionally, the chemical properties of the CESAs have been experimentally or computationally investigated, compared with known compounds and correlated with the observed staining behavior. Finally, to prove the potential of CECT-based 3D histopathology, we studied the effect of a cuprizone-induced demyelination model on myelin distribution throughout the brain. Based on the superior contrast-to-noise ratio between white and gray matter, we selected Hexabrix as a CESA.

## 2. Materials and methods

### 2.1. Sample preparation

Brains of nude mice (Rj:NMRIfire-*Foxn1*^nu/nu^, Janvier-Labs) were used to determine the staining kinetics, the effect of CESAs on tissue integrity and the contrast-to-noise ratios between white and gray matter for the four different CESAs. Brains of C57Bl/6JRj mice (male, 7 weeks old, Janvier-Labs) were used to investigate the affinity of the four CESAs towards different tissue constituents and for the cuprizone experiment.

#### 2.1.1. Nude mice

All animal procedures were performed in accordance with national and institutional guidelines for animal care. The brains (*n* = 6) were leftover tissues from another project that obtained the approval of the Ethical Committee for Animal Experiments, KU Leuven (ECD, project 160/2019). Mice were sacrificed by cervical dislocation, after which the brains were harvested and washed in phosphate-buffered saline (PBS, 10 mM, pH 7.41). Next, the dissected murine brains were fixed overnight using a neutral-buffered formalin solution (4% formaldehyde in PBS) at 4°C. After fixation, the brains were washed overnight in PBS at 4°C. Finally, the washing solution was exchanged with fresh PBS, in which the brains were stored at 4°C until the experiments started. Upon initiation of the experiments, the brains were cut in hemispheres (*n* = 12).

#### 2.1.2. C57B1/6JRj mice

All animal procedures were performed in accordance with national and institutional guidelines for animal care (Ethical committee: 2018/UCL/MD/024). Mice (*n* = 6 healthy, *n* = 2 cuprizone-treated) were fed for six weeks with either the control food [EF AIN93G – Growth (ssniff- E15712-04)] or food containing cuprizone [EF AIN93G + 0.2% Cuprizone (ssniff - S8899-E040)]. Each week, food pellets were replaced by fresh food. During treatment, the weight of each mouse was recorded as well as food intake per cage (Supplementary Table 2). After 6 weeks, the mice were anesthetized using isoflurane followed by decapitation. Cardiac perfusion was performed with a solution of 4% PFA in PBS. Following dissection, brains were immersed in 4% PFA at 4°C. After fixation, the brains were briefly washed in PBS at 4°C. Before starting the staining experiment, brains were cut in half (*n* = 12 healthy hemispheres, *n* = 3 cuprizone-treated hemispheres).

### 2.2. Contrast-enhancing staining agents

**FIGURE 1:**
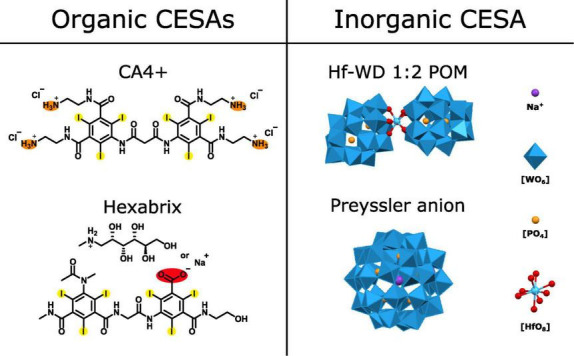
Schematic overview of the evaluated CESAs. Organic CESAs: CA4+ and Hexabrix [Ioxaglate anion with sodium or *N*-methyl-D-glucamine (NMDG) as counter cation]. Orange = positively charged functional group at physiological pH, red = negatively charged functional group at physiological pH and yellow = heavy atom. Inorganic CESAs: Hf-WD 1:2 POM, Preyssler anion and POM building blocks. Heavy atoms are Tungsten (W) and Hafnium (Hf).

A schematic representation of the investigated CESAs can be found in Figure 1. Since only Hexabrix^®^ ([C_24_H_20_I_6_N_5_O_8_]^–^, Guerbet, 320 mgI/ml, 39.3 m/V% Ioxaglate meglumine and 19.6 m/V% Ioxaglate sodium, anionic, iodinated contrast agent) has been commercially available and was available during the study, the other CESAs were synthesized in-house. CA4+ ([C_27_H_36_I_6_N_10_O_6_]^4 +^, cationic, iodinated contrast agent) was synthesized according to the protocol published by Stewart et al. (2017). The Hf-WD 1:2 POM (K_16_[Hf(α_2_-P_2_W_17_O_61_)_2_]⋅19H_2_O, anionic, tungsten-containing contrast agent) was synthesized according to the protocol published by Kato et al. (2006). Finally, the Preyssler anion (K_14_[NaP_5_W_30_O_110_]⋅31H_2_O, anionic, tungsten-containing contrast agent) was isolated as a side product from the synthesis of the Parent Wells-Dawson phosphotungstate (K_6_[α/β-P_2_W_18_O_62_]⋅14/19H_2_O) following the procedure available in the book Inorganic Syntheses. (Ginsberg, 1990) Purity of the synthesized compounds was confirmed by proton (^1^H) or phosphorus (^31^P) nuclear magnetic resonance (NMR) spectroscopy.

### 2.3. Preparation and properties of the staining solutions

The staining solutions for each CESA were prepared using PBS as the solvent. The respective concentrations and properties of the CESAs and staining solutions are listed in Table 1. Note that the solution of the Preyssler anion was never completely clear, and had a gray, cloudy appearance.

**TABLE 1:** Overview of the properties of the CESAs and their respective staining solutions.

CESA	Molecular weight (g/mol)	Heavy atom	Charge	Hydrodynamic radius (nm) – I	Hydrodynamic radius (nm) – V
CA4+	1500	Iodine (I)	+4	1.39 ± 0.50	0.985 ± 0.34
Hexabrix^®^	1291(Na^+^)/1463(NMDG^+^)	Iodine (I)	–1	1.46 ± 0.40	1.179 ± 0.34
Preyssler anion	8559	Tungsten (W)	–14	1.58 ± 0.36	1.369 ± 0.34
Hf-WD 1:2 POM	9473	Tungsten (W) and Hafnium (Hf)	–16	2.84 ± 0.70	2.474 ± 0.61
**CESA**	**CESA concentration (mmol/L)**	**CESA concentration (m/V% or V/V%)**	**pH of solution**	**Viscosity (mPa.s)**	**Density (g/cm^3^ or ml)**
CA4+	42	6.3 m/V%	6.87	1.0586 ± 0.0089	1.03740
Hexabrix^®^	169	40 V/V%	7.09	1.7145 ± 0.0081	1.15620
Preyssler anion	6.49	5 m/V%	6.74	0.9033 ± 0.0037	1.03517
Hf-WD 1:2 POM	3.69	3.5 m/V%	6.71	0.9042 ± 0.0037	1.03346

NMDG^+^, N-methyl-D-glucamine cation. Hydrodynamic radius (nm) – I = intensity-based and hydrodynamic radius (nm) – V = volume-based.

Dynamic viscosities were determined by rolling ball viscosity measurements at 25°C (Anton Paar Lovis 2000 M/Me rolling ball viscometer, Table 1). Prior to the measurements, all particles (e.g., precipitate, dust) were removed via centrifugation (Eppendorf Centrifuge 5810, Rotor A-4-44, 10 min, 4,000 rpm, 2,778 g).

The intensity- and volume-based particle size distribution (PSD) of the CESA solutions were determined by dynamic light scattering (DLS) at 25°C (Table 1). The backscattering of a 632.8 nm laser at 173° was measured using the Zetasizer Nano ZSP (Malvern Panalytical Ltd., Malvern, UK). Prior to data acquisition, all possible undesired particles (e.g., precipitate, dust) were removed from the solutions by centrifugation (15 min, 12,000 rpm, 13,800 *g*). The average PSD was determined from three consecutive measurements and derived from the DLS correlation function data (Supplementary Figure 1) by the software provided by the manufacturer (Zetasizer Software 7.03, Malvern, Paalytical Ltd., Malvern, UK). The hydrodynamic radii of the molecules in solution were corrected using the measured viscosities.

### 2.4. Staining procedure

**FIGURE 2:**
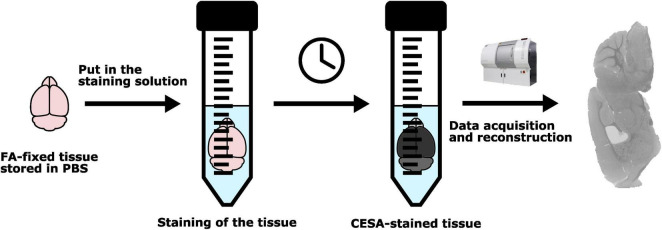
Schematic overview of the staining protocol.

A schematic overview of the staining procedure can be found in Figure 2. Information regarding the ratio of the staining volume with respect to the tissue volume can be found in Supplementary Figure 2.

#### 2.4.1. Nude mice hemispheres

The hemispheres were first gently dried on a piece of paper and transferred to Eppendorf tubes containing 5 ml of the staining solution. The Eppendorf tubes were incubated at room temperature while shaking for 1, 2, 4, and 9 days. Note that the Hf-WD 1:2 POM solution showed slight formation of a white precipitate upon prolonged staining (after 2 days).

#### 2.4.2. C57B1/6JRj mice hemispheres

The hemispheres were first gently dried on a piece of paper and transferred to Eppendorf tubes containing 5 ml of the staining solution. The healthy hemispheres were stained with all four CESAs and the cuprizone-treated hemispheres were stained with Hexabrix. The Eppendorf tubes were incubated at room temperature while shaking for 12 days to ensure full staining. Note that the Hf-WD 1:2 POM solution showed slight formation of a white precipitate upon prolonged staining (after 2 days).

### 2.5. CECT image acquisition and reconstruction

Samples were imaged using a Phoenix NanoTom M (GE Measurement and Control Solutions, Germany), with a diamond-coated tungsten target. During image acquisition, the samples were left in the Eppendorf tube, containing staining solution. For the kinetic experiment, each sample was imaged in fast scan mode at dedicated time points (1, 2, 4, and 9 days after immersion in the staining solution). After 9 days of staining, a normal mode image acquisition was performed on one representative hemisphere per CESA. For the cuprizone model experiment, normal mode image acquisition was performed after 12 days of staining. Detailed acquisition parameters can be found in Table 2. The reconstruction was performed using Datos|x GE Measurement and Control Solutions software (version 2.7.0 – RTM) where the image was cropped to reduce the size of the dataset and the inline median, ROI CT filter and Filter volume algorithms, implemented in the software, were applied. After reconstruction (16-bit Tiff), black (0) represents no attenuation and white (65535) represents the most attenuation.

**TABLE 2:** Overview of acquisition parameters.

Parameters	Fast acquisition mode	Normal mode	Normal mode cuprizone model
Focus-detector distance	250 mm	250 mm	250 mm
Focus-object distance	15 mm	15 mm	15 mm
Voxel size	6 μm	6 μm	6 μm
Tube voltage	60 kV	60 kV	60 kV
Tube current	420 μA	420 μA	420 μA
Focal spot size	5.97 μm	5.97 μm	5.97 μm
Mode	0	0	0
Filter	0.1 mm Al filter	0.1 mm Al filter	0.1 mm Al filter
Exposure time	500 ms	500 ms	500 ms
Number of images	2,000	2,400	2,000
Averaging	1	3	2
Skip	0	1	1
Scan duration	16 min 40 s	1 h 26 min 32 s	55 min 27 s

### 2.6. Image processing and analysis

#### 2.6.1. Automatic histogram windowing and normalization

One dataset per CESA of 16-bit TIFF files was converted to 8-bit bmp files with automatic histogram windowing. Subsequently gray value normalization was performed on all other datasets per CESA, with simultaneous conversion of 16-bit TIFF files to 8-bit BMP files. The references for the normalization were the Eppendorf tubes and air. Both operations were performed using in-house-developed Matlab (MATLAB R2021b) scripts (Maes, 2022).

#### 2.6.2. Total hemisphere volume analysis

Binarization of the total hemisphere volume was accomplished by using interactive thresholding, magic wand, brush and watershed segmentation tools, implemented in Avizo (version 2021.1, ThermoFisher).

#### 2.6.3. Penetration rate analysis

To consistently separate stained from unstained tissue, choosing a gray value threshold was imperative. For each CESA, a different threshold above which tissue was considered stained, was manually chosen. This threshold was determined based on the least attenuating part of the tissue after complete staining was achieved (i.e., no gray value difference between subsequent time points was observed). Gray values were measured using Fiji (Schindelin et al., 2012) and processed the JMP 16 Pro statistical software. Stained volumes were measured using interactive thresholding [Avizo (version 2021.1, ThermoFisher)].

**FIGURE 3:**
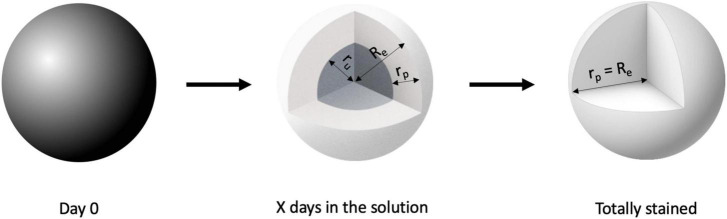
Visual representation of spherical approximation of the penetration depth. The spheres represent the staining process (Dark gray = unstained, white = stained) and graphically show the meaning of the radii.

We approximated the hemisphere as a sphere based on stained and total volume (Figure 3) and determined how the depth of the penetration front of the CESA through the sphere (r_p_) increased over time, until it is equal to the total radius of the sphere (R_e_), by using an exponential decay function (GraphPad Prism 9 [Version 9.3.1]).


(1) $$\begin{array}{c} R\,a\,d\,i\,u\,s\,o\,f\,t\,h\,e\,e\,n\,t\,i\,r\,e\,s\,p\,h\,e\,r\,e:R_{e}=\sqrt[{3}]{\frac{3}{4}\,\frac{V_{e}}{\pi }} \end{array}$$

(2) $$\begin{array}{c} R\,a\,d\,i\,u\,s\,o\,f\,t\,h\,e\,u\,n\,s\,t\,a\,i\,n\,e\,d\,s\,p\,h\,e\,r\,e:r_{u}=\sqrt[{3}]{\frac{3}{4}\,\frac{V_{u}}{\pi }} \end{array}$$

(3) $$\begin{array}{c} D\,e\,p\,t\,h\,o\,f\,C\,E\,S\,A\,p\,e\,n\,e\,t\,r\,a\,t\,i\,o\,n\,f\,r\,o\,n\,t:r_{p}=R_{e}-r_{u} \end{array}$$

With R*_*e*_* = radius of entire sphere, r*_*p*_* = depth of penetration of the CESA front, r*_*u*_* = radius of the unstained part of the sphere, V_u_ = unstained volume, and V_e_ = entire volume. The depth of penetration was determined using Eq. 3.

#### 2.6.4. Contrast-to-noise ratio

The contrast-to-noise ratio (CNR) was computed on the 16-bit TIFF files according to equation 4,


(4) $$\begin{array}{c} C\,N\,R=\frac{\left|\mu_{1}-\mu_{2}\right|}{\left(\frac{\sigma_{1}+\sigma_{2}}{2}\right)} \end{array}$$

where μ represents the mean gray value of the corpus callosum or cerebral cortex (most inner layer) in the dataset and σ represents the standard deviation on the mean gray value. Gray value measurements were performed using Fiji software (Schindelin et al., 2012).

#### 2.6.5. Structural analysis of cuprizone treated and untreated hemispheres

Gray value analysis: Note that all analyses were performed on the 16-bit Tiff files. All datasets contained two reference materials, a ceramic bead (Al_2_O_3_, G_B_) and the Eppendorf tube (G_E_). These were used to normalize the measured gray values via the following formula (Eq. 5):


(5) $$\begin{array}{c} g_{X,i}=\frac{G_{X,i}-G_{E,i}}{G_{B,i}-G_{E,i}} \end{array}$$

gray values were measured at four different positions in the corpus callosum region (caudal-medial, caudal-lateral, rostral-medial, and rostral-lateral) on 50 consecutive slices (300 μm).

Before initiating further analyses, all datasets were reoriented in Avizo using the “slice” module. Reorientation was chosen so that the coronal plane was aligned with the xy-plane.

##### 2.6.5.1. Fractional anisotropy analysis

A diffusion-tensor imaging (DTI)-like algorithm was applied, developed by NOVITOM.^1^ The algorithm, previously described by Chourrout et al. (2022a), was developed based on image gradient analysis to detect the orientation of white-matter fibers. We selected the following input parameters, adapted to the mouse brain: gradient orientation (0.5), gradient orientation tensor (0.5) and fiber orientation tensor (5). A spherical volume-of-interest (VOI) of 50 slices (300 μm) was used in the mid and rostral striatum region.

##### 2.6.5.2. Volume fraction analysis

To determine the volume fraction of myelinated fibers in the corpus striatum, Avizo was used. A VOI of 170 slices (1.02 mm) was created within the caudal striatum region. This VOI started 50 slices below the region where the first fibers were discernable. The volume fraction of myelinated fibers within these normalized images was determined using a manually selected gray value threshold that was constant between datasets.

##### 2.6.5.3. White matter fiber bundle thickness analysis

The cylinder correlation algorithm [Avizo (version 2021.1, ThermoFisher), parameters in Supplementary Table 3] was applied to the VOI selected for volume fraction determination. The “marker-based watershed inside mask” algorithm was used to label all fiber bundles individually. The Label analysis module was used to determine the thickness (Thickness3D) of the detected fiber bundles.

### 2.7. Brightfield microscopy

Classical 2D histological assessment was performed on a control murine hemisphere sample (formalin-fixed paraffin-embedded, no CESA staining), to serve as a reference, allowing validation and interpretation of the CECT images. This sample was embedded in paraffin wax and sectioned in 5 μm thin slices using a microtome (Microm 340E). The brain sections were colored with: Hematoxylin and Eosin Y (H&E), and luxol fast blue (LFB) and cresyl violet acetate (CV, Merck – C5042-10G) using standard protocols (*vide infra*). The stained tissue sections were scanned using a Leica SNC400 slide scanner.

#### 2.7.1. Haematoxylin and eosin Y staining

Paraffin sections (5 μm thick) were deparaffinized in three baths of toluol (5 min each) and three baths of isopropanol (5 min each), rinsed under running tap water (5 min) and rinsed in MilliQ water. Sections were stained for 10 min with Mayer’s haematoxylin staining solution (Merck – 1.04302), washed under running tap water (10 min) and rinsed using MilliQ water. The sections were subsequently put in ethanol (EtOH, 50%) and EtOH (70%) after which they were incubated with 1% alcoholic Eosin Y solution (Merck – 1.15935) for 2 min at room temperature, briefly rinsed in EtOH (70%) and EtOH (99–100%). Finally, the coverslip was mounted with mounting medium [Entellan New (Merck, 1.07961)].

#### 2.7.2. Luxol fast blue and cresyl violet staining

Paraffin sections (5 μm thick) were deparaffinized in three baths of toluol (5 min each) and three baths of isopropanol (5 min each). The sections were immersed in 0.1% Luxol Fast Blue solution (Hopkin and Williams, 016765) overnight at 60°C. Sections were cooled down to room temperature for 5 h. Sections were washed with EtOH (95%) for two times one min. Differentiation was performed by incubation with lithium carbonate (Li_2_CO_3_, 0.05%) and EtOH (70–90%), for 90 s each. The sections were differentiated by rinsing them twice with distilled water (10 s), visually inspected and differentiated again if needed. The sections were then stained with 1% CV solution for 15 min and washed three times with 100% EtOH for 3 min. Finally, the coverslip was mounted with mounting medium [Entellan New (Merck, 1.07961)].

### 2.8. Statistical analysis

Statistical tests were performed with GraphPad Prism 9 (version 9.3.1). For comparing two conditions, the unpaired *t*-test or multiple comparison tests (Tukey and Dunnett) were applied. For comparison of more than two conditions, we applied a One-Way ANOVA and Two-Way ANOVA. In the figure legends, the specific tests are mentioned, combined with the computed p-values and 95% confidence intervals. For all statistical tests, we used α = 0.05, not significant (no star): *p*-value > 0.05, *: *p*-value ≤ 0.05, ^**^: *p*-value < 0.01, ^***^: *p*-value < 0.001, ^****^: *p*-value < 0.0001.

## 3. Results

### 3.1. The chemical properties of the CESAs and their respective staining solutions determine the penetration rate through the tissue and effect on tissue volume

**FIGURE 4:**
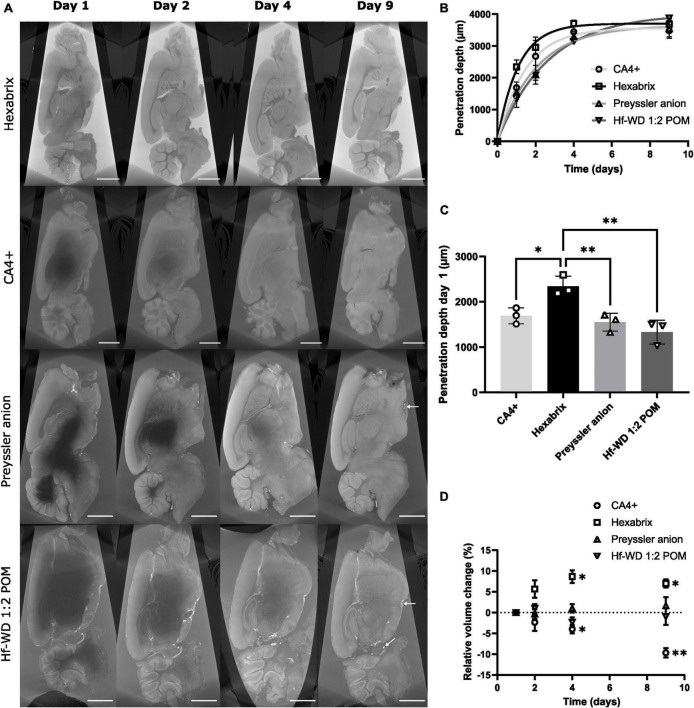
Penetration depth over time per CESA. **(A)** Typical CECT cross-sections of murine hemispheres that were stained with CA4+, Hexabrix, Preyssler anion or Hf-WD 1:2 POM imaged in fast acquisition mode after 1, 2, 4, and 9 days of staining (*n* = 3 per CESA) (scale bar = 1 mm). White arrows (Preyssler anion and Hf-WD 1:2 POM, day 9) indicate blood vessels (empty and filled). **(B)** Evolution of the approximated penetration radius over time (mean ± SD). Fitted exponential decay function: rp⁢(t)=Re-(Re⁢e*⁢x⁢p(-k⁢t)), with r_p_ = depth of penetration front, R_e_ = asymptote and k = growth rate. **(C)** Bar plot comparison between penetration depths after one day of staining (data points = individual samples, whiskers = standard deviation, *n* = 3). Ordinary One-Way ANOVA (α = 0.05, *p*-value = 0.0023) followed by Tukey’s multiple comparisons test (Hexabrix vs CA4+: *p*-value = 0.0243, 95%CI [0.0914, 1.22]; Hexabrix vs Preyssler anion: *p*-value = 0.0085, 95%CI [0.2308, 1.359]; Hexabrix vs. Hf-WD 1:2 POM: *p*-value = 0.0019, 95%CI [0.4495, 1.578]). **(D)** Relative total volume change over time during staining for each CESA (*n* = 3). Two-Way ANOVA followed by Dunnett’s multiple comparisons test vs day 1 (CA4+: Day 3: *p*-value = 0.0387, 95%CI [−0.498, −7.5]; Day 4: *p*-value = 0.0091, 95%CI [−5.62, −13.71], Hexabrix: Day 3: *p*-value = 0.019,6 95%CI [3.318, 14.02]; Day 4: *p*-value = 0.013, 95%CI [3.498, 10.50]).

To investigate the staining kinetics of the CESAs in the hemispheres, a time-lapsed staining experiment was performed (Figure 4). The approximated penetration depth of the CESAs in the hemisphere was modeled using an exponential decay function (Figure 4B). When the penetration radius increased, the concentration gradient gradually decreased in tandem with the penetration rate. To avoid the influence of approaching penetration fronts, we performed a comparison of the penetration depth after one day of staining (Figure 4C). This indicated that Hexabrix penetrates the tissue significantly faster compared to all other CESAs. No significant differences were observed between CA4+ and both polyoxometalates (POMs), but CA4+ seemed to be slightly faster. These observations were in line with the smaller hydrodynamic radii of the organic CESAs compared to the inorganic CESAs and the higher initial concentration gradient between the bulk solution and the tissue, both promoting faster diffusion through the tissue (Table 1).

The effect of the CESAs on the tissue integrity was also studied. The organic CESAs consistently induced changes in the total hemisphere volume, with CA4+ causing shrinkage (−9.7 ± 1.2%) and Hexabrix causing swelling (+7.0 ± 1.0%), while no influence was observed for the inorganic CESAs (Figure 4D). Viscosity measurements of the stock solutions showed that the organic CESAs (Hexabrix in particular) had a tendency to alter the viscosity, while this was not the case for the inorganic CESAs (Table 1). As a result, interactions between the solvent and CESA molecules could be correlated with swelling or shrinkage of the tissue.

### 3.2. CECT enables to study the microanatomy of the murine brain tissue

**FIGURE 5:**
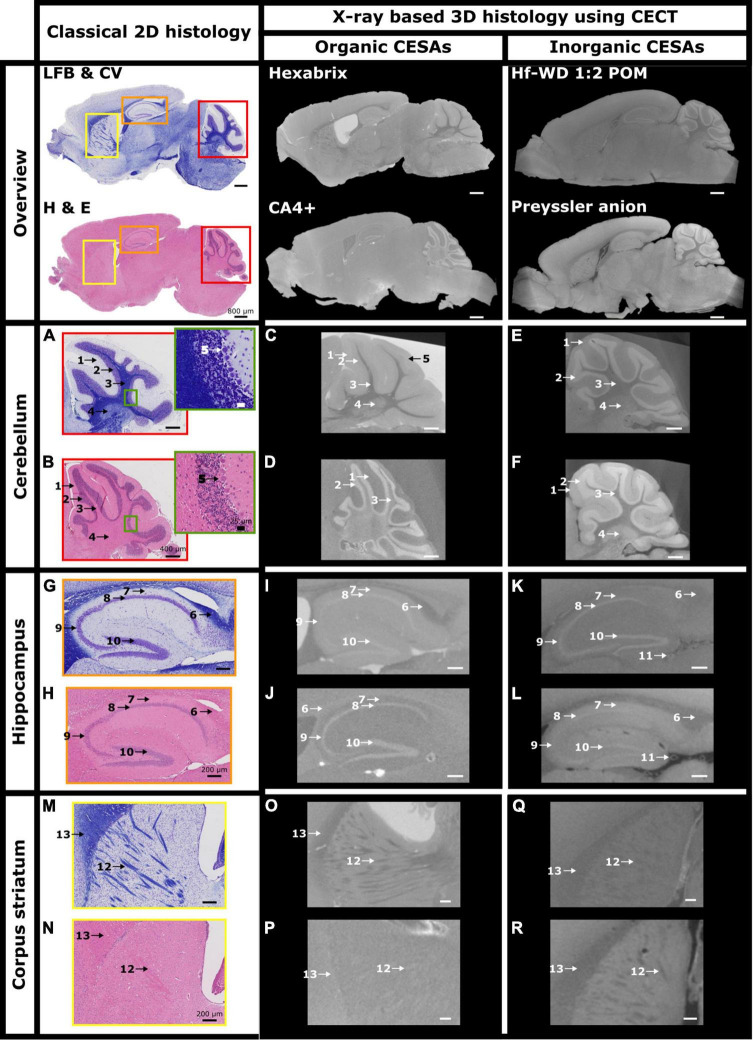
Investigation of CESA distribution throughout the murine hemisphere. Overview and zoom on cerebellum, hippocampus and corpus striatum of cross-sections of murine hemispheres stained by LFB & CV **(A,G,M)**, H&E **(B,H,N)**, Hexabrix **(C,I,O)**, CA4+ **(D,J,P)**, Hf-WD 1:2 POM **(E,K,Q)**, and Preyssler anion **(F,L,R)**. 1 = molecular layer, 2 = granular layer, 3 = white matter, 4 = cerebellar nuclei, 5 = Purkinje cell layer, 6 = corpus callosum, 7 = stratum oriens, 8 = stratum pyramidale (CA1), 9 = stratum pyramidale (CA2 and/or CA3), 10 = stratum granulare (Dentate gyrus), 11 = blood vessel, 12 = Striatopallidal fiber bundles, 13 = corpus callosum.

The anatomical analysis of CECT slices was performed with the help of the book “Neuroanatomy of the mouse, an introduction” as well as LFB&CV- and H&E-stained sections (Schröder et al., 2020). The CESAs had different affinities for the cerebellum, the hippocampus and striatum region (Figure 5, red, orange, and yellow regions, respectively, Supplementary Videos 1–4). Within the cerebellum, Hexabrix staining resulted in a clear contrast difference between the white matter (Figure 5C: 3), gray matter (Figure 5C: 1, 2 and 5) and cerebellar nuclei (Figure 5C: 4). Furthermore, this was the only CESA that allowed the identification of the Purkinje cell layer (Figure 5C: 5) as a slightly hyperintense region between the granular (Figure 5C: 2) and molecular (Figure 5C: 1) layer. The granular layer was found to be slightly hypointense compared to the molecular layer. For CA4+ staining, distinction between the white matter and cerebellar nuclei was not possible (Figure 5D: 3). Nonetheless, there was an excellent contrast between the granular layer (Figure 5D: 2, hyperintense) and the surrounding structures (Figure 5D: 1 and 3). For both POMs, the white matter (Figures 5E, F: 3) showed the lowest gray value, followed by the molecular layer (Figures 5E, F: 1 and 4) and finally the granular layer (Figures 5E, F: 2). In both cases, we could distinguish between the cerebellar nuclei and the white matter (Figures 5E, F: 3 and 4).

In the hippocampus, Hexabrix staining again enabled to differentiate between white matter (Figure 5I: 6) and surrounding tissue. In addition, the layers containing the pyramidal (Figure 5I: 8 an 9) and granule cells (Figure 5I: 10) within Ammon’s horn (CA) and the dentate gyrus (DG), respectively, were visible (slightly hyperintense signals). CA4+ staining showed a weaker contrast between the corpus callosum (Figure 5J: 6) and the surrounding tissues compared to Hexabrix. The layers containing the pyramidal and granule cell layers of CA and DG, respectively, were clearly delineated. Interestingly, different staining behavior between both POMs was observed. Hf-WD 1:2 POM staining did not provide a clear contrast between the corpus callosum (Figure 5K: 6) and surrounding tissue, but was able to highlight the pyramidal (Figure 5K: 8 and 9) and granule cell (Figure 5K: 10) layers of the CA and DG, respectively. Preyssler anion provided a clear contrast between the corpus callosum (Figure 5L: 6) and surrounding tissues, but it was harder to discern the different layers of the hippocampus. Additionally, both POMs show affinity towards empty (Figures 5K, L: 11) and filled blood vessels (Figure 4A: white arrows), which fill in correctly with good ref was not the case for the organic CESAs. For the striatum region, staining with Hexabrix and both POMs showed the striatopallidal fibers as hypointense (Figures 5O, Q, R: 12) compared to the surrounding tissue, while CA4+ showed them as weakly hyperintense (Figure 5P: 12).

**FIGURE 6:**
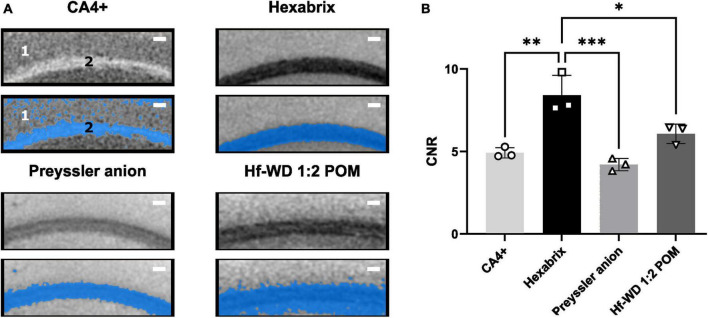
Comparison of ease of segmentation per CESA based on CNR. **(A)** Illustration of the effect of the ease of binarization between white matter (corpus Callosum) and gray matter (cerebral cortex) using automatic thresholding (Otsu criterion). 1 = Cerebral cortex, 2 = Corpus callosum. Scale bar = 100 μm. **(B)** CNR values for gray and white matter comparison for all CESAs. Ordinary One-Way ANOVA (α = 0.05, *p*-value = 0.0005) followed by Tukey’s multiple comparisons test (*n* = 3, Hexabrix vs CA4+: *p*-value = 0.0015, 95%CI [1.619, 5.356]; Hexabrix vs. Preyssler anion: *p*-value = 0.0004, 95%CI [2.331, 6.068]; Hexabrix vs. Hf-WD 1:2 POM: *p*-value = 0.0165, 95%CI [0.4702, 4.207]).

The contrast-to-noise ratio (CNR) between white (corpus callosum) and gray (cerebral cortex) matter was determined as quantitative measure for the contrast difference and ease of segmentation between these structures (Figure 6). This indicated that Hexabrix staining resulted in the highest contrast between gray and white matter (Figure 6B). This was confirmed by a clean automatic thresholding-based binarization (Figure 6A).

### 3.3. Hexabrix staining allows the detection of cuprizone-induced demyelination

**FIGURE 7:**
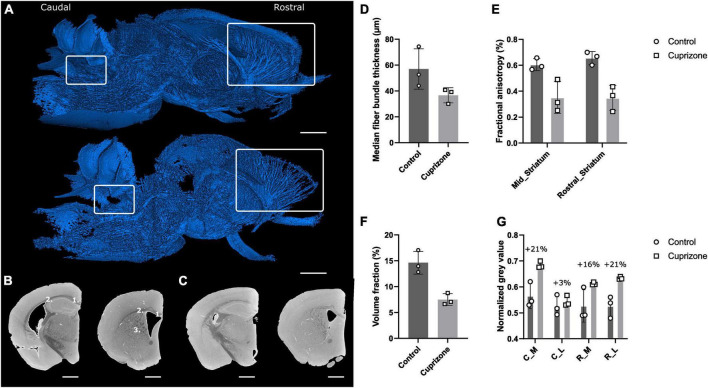
Effect of cuprizone treatment on myelination using Hexabrix staining and quantitative white matter parameters. **(A)** 3D rendering of segmented white matter of a cuprizone-treated hemisphere (top) and a healthy hemisphere (bottom). White rectangles indicate regions where demyelination was expected (left: white matter near cerebellar nuclei, right: corpus callosum and corpus striatum). **(B)** Caudal (left) and rostral (right) coronal gray scale cross-sections of a healthy hemisphere with medial (1) and lateral (2) corpus callosum and corpus striatum (3). Scale bar = 1 mm. **(C)** Caudal (left) and rostral (right) coronal gray scale cross-sections of a cuprizone-treated hemisphere. Scale bar = 1 mm. **(D)** Median fiber bundle thickness of healthy and cuprizone-treated hemispheres. **(E)** Computed fractional anisotropy of healthy and cuprizone-treated hemispheres on the rostral part of the striatum and the middle part of the striatum. **(F)** Volume fraction of white matter fiber bundles in the striatum. **(G)** Normalized gray values of healthy and cuprizone-treated corpus callosum. C_M, caudal, medial; C_L, caudal, lateral; R_M, rostral, medial; R_L, rostral, lateral.

Based on the superior contrast between gray and white matter after Hexabrix staining, we decided to apply this CESA to a cuprizone-induced demyelination model to evaluate the potential of this CESA as a new probe for monitoring of myelin loss and consequently, remyelination (Figure 7 and Supplementary Video 5). Briefly, cuprizone treatment is known to induce demyelination in the white matter by inducing the death of oligodendrocytes in specific locations within the brain (Zirngibl et al., 2022). The effectiveness of the cuprizone diet was confirmed by loss of appetite and weight during the treatment period (Supplementary Table 2 and Supplementary Figure 3) (Babbs et al., 2020).

Qualitatively, the impact of demyelination was obvious when comparing 3D renderings of the binarized white matter using a manually selected, normalized gray value threshold (Figure 7A). The 3D renderings allowed spatial assessment of demyelination, in contrast with conventional 2D histological-based assessment, which often is limited to several sections in predefined regions (e.g., rostral vs caudal) (Xie et al., 2010). The cerebellum white matter seemed only slightly affected near the cerebellar nuclei region, while the corpus striatum and corpus callosum regions were strongly affected by the cuprizone-induced demyelination. Moreover, the 3D renderings showed that also other regions (e.g., brainstem white matter fiber bundles) seemed affected. Since myelin repels Hexabrix, demyelinated regions were characterized by increased gray values, due to higher concentrations of Hexabrix. Comparison of gray scale cross-sections of the rostral and caudal corpus striatum and corpus callosum confirmed an increase in gray value where demyelination was expected (Figures 7B, C).

Quantitative analyses of thickness, gray value-based fractional anisotropy and volume fraction of myelinated white matter fiber bundles and gray values measurements of the corpus callosum supported the qualitative observations. Both thickness and volume fraction of myelinated white matter fiber bundles were decreased after cuprizone-induced demyelination (Figures 7D, F). Given that binarization was carried out using a normalized gray value threshold based on the gray values of healthy tissue, this suggested reduced myelination of the white matter fibers. The gray value-based fractional anisotropy (diffusion tensor imaging (DTI)-like parameter, extracted from image gradient analysis) was decreased in the mid and rostral striatum, which is consistent with a weaker contrast between the white matter fiber bundles and the background gray matter (Figure 7E). Furthermore, the radial diffusivity (DTI-like) was increased (data not shown). Finally, gray value measurements in the rostral and caudal corpus callosum revealed caudal-rostral and medio-lateral effects of cuprizone treatment (Figure 7G). The highest percentages of demyelination were observed for the rostral-lateral and caudal-medial corpus callosum, whereas a small increase was observed for the caudal-lateral corpus callosum.

## 4. Discussion

To aid in unraveling the structure-function relationship of the brain, high-resolution 3D imaging of the brain microstructure is required. Within the field of *ex vivo* brain CECT imaging, a plethora of CESAs have already been evaluated (Supplementary Table 1). While some staining mechanisms have been proposed, no systematic approach has been used to screen different (physico)chemical parameters. In this work, four CESAs that are new to the field of brain CECT imaging are presented. With the intention of connecting their chemical properties to their staining behavior, the CESAs were selected to minimize molecular variance within each set. This knowledge is highly valuable for the study and design of future candidate CESAs for brain imaging using CECT.

We found that the organic CESAs penetrated the hemisphere faster than the inorganic CESAs. This was correlated with both the initial concentration gradient between the bulk solution and the tissue, and with the hydrodynamic radii of the molecules in solution. Both parameters impact diffusion of the molecules into the tissue via different mechanisms. The concentration gradient will promote diffusiophoretic processes (Velegol et al., 2016), whereas the hydrodynamic radii are correlated with the Brownian motion of the molecules in solution (Miller, 1924; Maguire et al., 2018). However, for the inorganic CESAs, the hydrodynamic radii combined with their initial concentrations might seem to contradict their similar penetration rate. Nonetheless, it is known that the Hf-WD 1:2 POM will likely dissociate in smaller POM species (Vandebroek et al., 2018) during the staining process. These have smaller, more similar hydrodynamic radii compared to Preyssler anion. Next, while it might seem that the initial concentration gradients give an advantage to the organic CESAs, the inorganic CESAs are not able to reach such high concentrations and are showing signs of precipitation at their current concentration. Hence, the differences in initial concentration gradient are linked to the inherent solubility profiles of the evaluated CESAs. For this, determination of the total solubility (mmol heavy atom/L) of CESAs would be a valuable parameter for further design of staining protocols. It should be noted that a lower concentration of POMs does not necessarily impact the quality of the image, since the heavy atoms of which the POMs are composed (_74_W and _72_Hf) attenuate X-rays more strongly than the heavy atom in the organic CESAs (_53_I).

In addition to differences in initial concentration gradient and hydrodynamic radii between the CESAs, molecular interactions of the CESAs with the tissue constituents will also influence the penetration rate (Kusova et al., 2021) of the CESAs through the tissue. Note that the description of the affinity depends on the smallest features we can distinguish and hence on the spatial and contrast resolution. A rule of thumb applied to CECT images dictates that the true spatial resolution often equals two to three times the voxel size (Rawson et al., 2020). In our case, this would mean that the true spatial resolution is between 12 and 18 μm. All evaluated CESAs had different affinity profiles towards different microanatomical regions in the highly heterogeneous brain environment. However, the differences in affinity profiles were most pronounced within the set of the organic CESAs. These affinities are correlated with both the chemical properties of the CESAs and the tissue as well as the microstructure of the tissue. In the case of the organic CESAs, the main difference in chemical properties is their charge at pH 7.4. In this environment, Ioxaglic acid (Hexabrix) is negatively charged and hence will be repelled by the myelin-rich brain structures. These consist out of approximately 70% lipid molecules, including sulfated and phosphorylated compounds (e.g., sulfatides, polyphosphoinositides, plasmalogens, lecithin, etc.), being negatively charged at physiological pH (Siegel, 2006). Comparison with known organic, iodinated CESAs suggests that negatively charged [e.g., Hypaque-76 (de Crespigny et al., 2008)] and uncharged but hydrophilic [e.g., Iopamiron (Saito and Murase, 2012; Udagawa et al., 2019)] molecules are both repelled by white matter. Basic calculated physicochemical properties (Supplementary Table 4, Percepta, ACD/Labs^®^) show that all CESAs are hydrophilic, but only CA4+ has a positive charge at physiological pH. Therefore, CA4+ will be attracted to white matter-rich structures as a result of electrostatic interactions. Moreover, a patented structure of a myelin-specific fluorescent dye (Kilgore, 2006) that should resemble the well-known FluoroMyelin Red fluorescent dye (Monsma and Brown, 2012), contains permanent positive charges and is known to preferentially interact with white matter. Based on these results and the literature, we conclude that within this set of organic CESAs, a positive charge is required for preferred interaction with white matter, whereas no charge, but hydrophilic, or a negative charge is linked to a repelling effect of white matter. Furthermore, interaction of CA4+ with white matter components will slow down its diffusion through the tissue, while this will not be the case for Hexabrix. Future research on the diffusion through the spinal cord, where white matter surrounds gray matter, would be important given the opposite affinity of both CESAs for white matter.

Within the set of the inorganic CESAs, the affinities towards different constituents were similar, except for the hippocampus. Hence, their affinity patterns should also not cause different penetration rates through the tissue. In general, POMs are known to have affinity for proteins (e.g., present in plasma) and other biomolecules (Van Rompuy and Parac-Vogt, 2019; Gil and Carbo, 2022), omnipresent in the fixed tissue, which explains their capability of revealing empty and filled blood vessels (Figures 4A, 5K, L: 11). However, none of the aforementioned interactions for both sets of CESAs can explain the affinity pattern towards anatomical features of high cell density (i.e., the granular layer of the cerebellum and the pyramidal and granular layers of CA and DG). Therefore, we conclude that next to differences in (non-) covalent molecular interactions between both sets of CESAs, also cell density, type and in general the tissue architecture drive the distribution of the CESAs within the tissue.

Apart from the penetration rate, we also evaluated the influence of CESA staining on tissue volume. The organic CESAs influenced the tissue volume, whereas this was not the case for the inorganic CESAs. Within this study, we tried to correlate this with the viscosity of the solution. The viscosity gives information about the inter- and intramolecular interactions between the CESAs and solvent. Measured viscosities are in line with the fact that the inorganic polyoxoanions might exhibit a weak tendency to interact with water molecules. Misra et al. (2020), Gil and Carbo (2022) Despite its correlation with the final tissue volume, the viscosity of the solution is unable to explain the opposite effect of both organic CESAs. Nonetheless, the swelling induced in the Hexabrix-stained samples might be due to the presence of the highly polar *N*-methyl-D-glucamine counter cation. This hygroscopic cation will also diffuse into the tissue during the staining process. The shrinkage induced by CA4+ could be related to the osmolality of the solution, considering that this molecule contains four counteranions, while Hexabrix only has one (Susaki et al., 2020). Finally, even though volume changes were induced by the organic CESAs ([−11%, +8%]), these volume changes can still be considered relatively small compared to volume changes induced by ethanol dehydration, PTA (−27.3% ± 1.7%) staining or iodine staining in aqueous (−38.8% ± 2%) or organic solvents (−66% ± 3.4%) (Buytaert et al., 2014).

To validate the applicability of CECT to investigate myelination in animal models, we selected the CESA with the highest contrast between gray and white matter and we applied it to a cuprizone-induced demyelination model. In practice, this animal model is one of the models used to study multiple sclerosis and new remyelinating treatments (Torkildsen et al., 2008). Hexabrix was used, based on the superior CNR obtained between white and gray matter, but this choice does not exclude the potential of other CESAs for such models. The effects of the cuprizone treatment on the staining behavior of Hexabrix did not only reaffirm the strengths of CECT-based 3D histopathology, but also validated the key role of myelin in the distribution of Hexabrix molecules through the tissue. Our methodology allowed quantitative 3D analysis of myelination in a whole murine hemisphere by investigating different metrics (DTI-like parameters, normalized gray values, fiber bundle thickness). In addition, compared to conventional 2D histology techniques, it offers a simple tool for studying the myelination of a specimen in 3D, high-resolution, in short timeframes and in any orientation. This experiment paves the way for applying CECT to more pathologies inducing changes in myelin distribution (e.g., central pontine myelinolysis) (Love, 2006). However, notice that an important limitation of this study is the small sample size that was used to evaluate the cuprizone treatment.

Depending on the research question, the appropriate choice of CESA should be made. None of the CESAs evaluated in this study is currently commercially available, except for a potential alternative for Hexabrix, called Telebrix^®^. Nonetheless, syntheses for all other CESAs are available. Throughout experiments, it was found that CA4+ staining was most variable in terms of the normalized gray values, while this was not the case for the other CESAs. To aid researchers in choosing the appropriate CESA for their experiment, we summarized key factors to consider when planning the experiment (Table 3).

**TABLE 3:** Summary of certain key factors to consider when applying the evaluated CESAs.

	Hexabrix	CA4+	Hf-WD 1:2 POM	Preyssler anion
Commercial availability	**−**	**−**	**−**	**−**
Synthesis protocols	**−**	**+**	**+**	**±**
Ease of synthesis	**NA**	**±**	**+**	**−**
Staining robustness	**+**	** ±**	**+**	**+**
CNR gray and white matter	**++**	**+**	**+**	**+**
Visualization cerebellar layers	**±**	**+**	**++**	**++**
Visualization vasculature	**−**	**−**	**+**	**+**
Tissue volume integrity	**−**	**−**	**+**	**+**
Penetration rate	**++**	**+**	**−**	**−**
Concentration required	**−**	**−**	**+**	**+**

## 5. Conclusion

We introduced four CESAs of low toxicity, new to the field of *ex vivo* brain CECT and limited in destructiveness for the tissue compared to known CESAs. All CESAs were able to completely stain a murine hemisphere within nine days. Furthermore, by evaluating two sets of chemically related molecules, we were able to provide specific information concerning the staining kinetics (i.e., hydrodynamic radius, concentration, viscosity) and affinities (i.e., molecular charge, cell density and type) of the CESAs towards tissue constituents. Finally, based on our acquired knowledge on the interactions of Hexabrix with myelin, we could correctly confirm an expected change in gray value due to cuprizone-induced demyelination. Nonetheless, the staining process remains extremely complex and future research is required to increase our understanding of diffusion kinetics and interaction mechanisms. Ultimately, this research will not only prove to be crucial for further development of CESAs for *ex vivo* brain CECT and 3D histopathology, but also for all other imaging modalities that make use of contrast agents that require interactions for differential staining of tissue constituents.

## Data availability statement

The original contributions presented in this study are included in the article/Supplementary material, further inquiries can be directed to the corresponding author.

## Ethics statement

This animal study was reviewed and approved by the Ethical Committee for Animal Experiments, KU Leuven (ECD, project 160/2019) and the University Hospital Woluwé, Saint-Luc Ethical Committee (2018/UCL/MD/024).

## Author contributions

TB, CP, FC, DH, WD, and GK contributed to the conceptualization and design of the study. TB and CP contributed to the data acquisition. TB, CP, and MC contributed to the data analysis. AM and AD treated and provided the mice used in the cuprizone model experiment. LV provided the Preyssler anion CESA and insights concerning the polyoxometalates. TB, CP, WD, and GK wrote the first draft of the manuscript. TB performed the chemical and statistical analyses. AD and FC provided insights concerning the cuprizone model. All authors contributed to manuscript revision, read, and approved the submitted version.

## Footnotes

1
https://www.novitom.com/en/

## References

[B1] Ahrens M. Orger M. Robson D. Li J. Keller P. (2013). Whole-brain functional imaging at cellular resolution using light-sheet microscopy. *Nat. Methods* 10 413–420. 23524393 10.1038/nmeth.2434

[B2] Anderson R. Maga A. M. (2015). A Novel procedure for rapid imaging of adult mouse brains with microCT using iodine-based contrast. *PLoS One* 10:e0142974. 10.1371/journal.pone.0142974 26571123 PMC4646620

[B3] Babbs R. Beierle J. Yao E. Kelliher J. Medeiros A. Anandakumar J. (2020). The effect of the demyelinating agent cuprizone on binge-like eating of sweetened palatable food in female and male C57BL/6 substrains. *Appetite* 150:104678. 10.1016/j.appet.2020.104678 32209386 PMC7206526

[B4] Baer K. Kieser S. Schon B. Rajendran K. Ten Harkel T. Ramyar M. (2021). Spectral CT imaging of human osteoarthritic cartilage via quantitative assessment of glycosaminoglycan content using multiple contrast agents. *APL Bioeng.* 5:026101. 10.1063/5.0035312 33834156 PMC8018795

[B5] Balint R. Lowe T. Shearer T. (2016). Optimal contrast agent staining of ligaments and tendons for X-Ray computed tomography. *PLoS One* 11:e0153552. 10.1371/journal.pone.0153552 27078030 PMC4831740

[B6] Barbone G. Bravin A. Mittone A. Grosu S. Ricke J. Cavaletti G. (2021). High-spatial-resolution three-dimensional imaging of human spinal cord and column anatomy with postmortem X-ray phase-contrast micro-CT. *Radiology* 298 135–146. 10.1148/radiol.2020201622 33107800

[B7] Batista-Garcia-Ramo K. Fernandez-Verdecia C. (2018). What we know about the brain structure-function relationship. *Behav. Sci.* 8:39. 10.3390/bs8040039 PMC5946098 29670045

[B8] Boretius S. Kasper L. Tammer R. Michaelis T. Frahm J. (2009). MRI of cellular layers in mouse brain in vivo. *Neuroimage* 47 1252–1260. 19520174 10.1016/j.neuroimage.2009.05.095

[B9] Buytaert J. Goyens J. De Greef D. Aerts P. Dirckx J. (2014). Volume shrinkage of bone, brain and muscle tissue in sample preparation for micro-CT and light sheet fluorescence microscopy (LSFM). *Microsc. Microanal.* 20 1208–1217. 10.1017/S1431927614001329 24963987

[B10] Chen K. Arad A. Song Z. Croaker D. (2018). High-definition neural visualization of rodent brain using micro-CT scanning and non-local-means processing. *BMC Med. Imaging* 18:38. 10.1186/s12880-018-0280-6 30376825 PMC6208172

[B11] Choi J. Yang X. Foley M. Wang X. Zheng X. (2017). Induction and micro-CT imaging of cerebral cavernous malformations in mouse model. *J. Vis. Exp.* 127:56476. 10.3791/56476 28892037 PMC5752174

[B12] Chourrout M. Rositi H. Ong E. Hubert V. Paccalet A. Foucault L. (2022a). Brain virtual histology with X-ray phase-contrast tomography Part I: Whole-brain myelin mapping in white-matter injury models. *Biomed. Optics Express* 13 1620–1639. 10.1364/BOE.438832 35415001 PMC8973191

[B13] Chourrout M. Roux M. Boisvert C. Gislard C. Legland D. Arganda-Carreras I. (2022b). Brain virtual histology with X-ray phase-contrast tomography Part II: 3D morphologies of amyloid-beta plaques in Alzheimer’s disease models. *Biomed. Optics Express* 13 1640–1653. 10.1364/BOE.438890 35414980 PMC8973161

[B14] Chuang N. Mori S. Yamamoto A. Jiang H. Ye X. Xu X. (2011). An MRI-based atlas and database of the developing mouse brain. *Neuroimage* 54 80–89. 10.1016/j.neuroimage.2010.07.043 20656042 PMC2962762

[B15] Dawood Y. Hagoort J. Siadari B. Ruijter J. Gunst Q. Lobe N. (2022). Reducing soft-tissue shrinkage artefacts caused by staining with Lugol’s solution. *Sci. Rep.* 12:2366. 10.1038/s41598-022-06160-4 PMC8821669 35132156

[B16] de Bournonville S. Vangrunderbeeck S. Ly H. Geeroms C. De Borggraeve W. Parac-Vogt T. (2020). Exploring polyoxometalates as non-destructive staining agents for contrast-enhanced microfocus computed tomography of biological tissues. *Acta Biomater.* 105 253–262. 10.1016/j.actbio.2020.01.038 31996331

[B17] de Crespigny A. Bou-Reslan H. Nishimura M. Phillips H. Carano R. D’Arceuil H. (2008). 3D micro-CT imaging of the postmortem brain. *J. Neurosci. Methods* 171 207–213. 18462802 10.1016/j.jneumeth.2008.03.006 PMC2693019

[B18] Feldman K. O’Keefe Y. Gignac P. O’Brien H. (2022). Highest resolution microCT scan of the human brainstem reveals putative anatomical basis for infrequency of medial medullary syndrome. *Neuroimage Clin.* 36:103272. 10.1016/j.nicl.2022.103272 36451373 PMC9723294

[B19] Gil A. Carbo J. (2022). Computational modelling of the interactions between polyoxometalates and biological systems. *Front. Chem.* 10:876630. 10.3389/fchem.2022.876630 35494630 PMC9046717

[B20] Ginsberg A. (1990). *Inorganic syntheses.* New York, NY: Wiley.

[B21] Heimel P. Swiadek N. Slezak P. Kerbl M. Schneider C. Nurnberger S. (2019). Iodine-enhanced micro-CT imaging of soft tissue on the example of peripheral nerve regeneration. *Contrast Media Mol. Imaging* 2019:7483745. 10.1155/2019/7483745 31049044 PMC6458925

[B22] Herrera M. Notario B. Barrio M. Metscher B. Murillo Gonzalez J. (2018). X-ray micro-computed tomography of postmortem brain tissue using potassium dichromate as a contrast agent. *Arch. Ital. Biol.* 156 48–53. 10.12871/00039829201815 30039835

[B23] Hillman E. Voleti V. Li W. Yu H. (2019). Light-sheet microscopy in neuroscience. *Annu. Rev. Neurosci.* 42 295–313. 31283896 10.1146/annurev-neuro-070918-050357 PMC6800245

[B24] Horobin R. (1975). Biological stains and their uses. *J. Soc. Dyers Colour* 91 4–14.

[B25] Ishiguchi T. Takahashi S. (2010). Safety of gadoterate meglumine (Gd-DOTA) as a contrast agent for magnetic resonance imaging. *Drugs R D* 10 133–145. 20945944 10.2165/11539140-000000000-00000 PMC3586093

[B26] Karhula S. Finnilä M. Freedman J. Kauppinen S. Valkealahti M. Lehenkari P. (2017). Micro-scale distribution of CA4+ in ex vivo human articular cartilage detected with contrast-enhanced micro-computed tomography imaging. *Front. Phys.* 5:538. 10.3389/fphy.2017.00038

[B27] Kato C. Shinohara A. Hayashi K. Nomiya K. (2006). Syntheses and X-ray crystal structures of zirconium(IV) and hafnium(IV) complexes containing monovacant wells-Dawson and Keggin polyoxotungstates. *Inorg. Chem.* 45 8108–8119. 10.1021/ic060656e 16999408

[B28] Kavkova M. Zikmund T. Kala A. Salplachta J. Proskauer Pena S. Kaiser J. (2021). Contrast enhanced X-ray computed tomography imaging of amyloid plaques in Alzheimer disease rat model on lab based micro CT system. *Sci. Rep.* 11:5999. 10.1038/s41598-021-84579-x 33727592 PMC7966753

[B29] Kerckhofs G. Sainz J. Marechal M. Wevers M. Van de Putte T. Geris L. (2014). Contrast-enhanced nanofocus X-Ray computed tomography allows virtual three-dimensional histopathology and morphometric analysis of osteoarthritis in small animal models. *Cartilage* 5 55–65. 10.1177/1947603513501175 26069685 PMC4297096

[B30] Kerckhofs G. Stegen S. van Gastel N. Sap A. Falgayrac G. Penel G. (2018). Simultaneous three-dimensional visualization of mineralized and soft skeletal tissues by a novel microCT contrast agent with polyoxometalate structure. *Biomaterials* 159 1–12. 10.1016/j.biomaterials.2017.12.016 29306094

[B31] Kilgore J. J. (2006). Lipophilic dyes and their application for detection of myelin. US 2006/0073541 A1. United States Patent Application Publication.

[B32] Koc M. Aslan N. Kao A. Barber A. (2019). Evaluation of X-ray tomography contrast agents: A review of production, protocols, and biological applications. *Microsc. Res. Techniq.* 82 812–848. 10.1002/jemt.23225 30786098

[B33] Kusova A. Sitnitsky A. Zuev Y. (2021). Impact of intermolecular attraction and repulsion on molecular diffusion and virial coefficients of spheroidal and rod-shaped proteins. *J. Mol. Liq.* 323:114927.

[B34] Llambrich S. Wouters J. Himmelreich U. Dierssen M. Sharpe J. Gsell W. (2020). ViceCT and whiceCT for simultaneous high-resolution visualization of craniofacial, brain and ventricular anatomy from micro-computed tomography. *Sci. Rep.* 10:18772. 10.1038/s41598-020-75720-3 33128010 PMC7599226

[B35] Lombardi S. Scola E. Ippolito D. Zambelli V. Botta G. Cuttin S. (2019). Micro-computed tomography: A new diagnostic tool in postmortem assessment of brain anatomy in small fetuses. *Neuroradiology* 61 737–746. 10.1007/s00234-019-02168-2 30693410

[B36] Longo D. Kasper D. Jameson J. Larry F. Hauser S. Loscalzo J. (2012). *Harrison’s principles of internal medicine.* New York, NY: McGraw Hill.

[B37] Love S. (2006). Demyelinating diseases. *J. Clin. Pathol.* 59 1151–1159. 17071802 10.1136/jcp.2005.031195 PMC1860500

[B38] Maes A. (2022). *Contrast-team/histogram-windowing: Initial release (v1.0). Zenodo.*

[B39] Maguire C. Rosslein M. Wick P. Prina-Mello A. (2018). Characterisation of particles in solution - a perspective on light scattering and comparative technologies. *Sci. Technol. Adv. Mat.* 19 732–745. 10.1080/14686996.2018.1517587 30369998 PMC6201793

[B40] Masis J. Mankus D. Wolff S. Guitchounts G. Joesch M. Cox D. D. (2018). A micro-CT-based method for quantitative brain lesion characterization and electrode localization. *Sci. Rep.* 8:5184. 10.1038/s41598-018-23247-z 29581439 PMC5980003

[B41] McInnes E. (2005). Artefacts in histopathology. *Comp. Clin. Pathol.* 13 100–108.

[B42] Miller C. (1924). The Stokes-Einstein law for diffusion in solution. *Proc. R. Soc. Lond. A* 106 724–749.

[B43] Misra A. Kozma K. Streb C. Nyman M. (2020). Beyond charge balance: Counter-cations in polyoxometalate chemistry. *Angew Chem. Int. Ed. Engl.* 59 596–612. 10.1002/anie.201905600 31260159 PMC6972580

[B44] Mizutani R. Takeuchi A. Uesugi K. Takekoshi S. Osamura R. Suzuki Y. (2010). Microtomographic analysis of neuronal circuits of human brain. *Cereb. Cortex* 20 1739–1748. 10.1093/cercor/bhp237 19915092

[B45] Monsma P. Brown A. (2012). FluoroMyelin (TM) red is a bright, photostable and non-toxic fluorescent stain for live imaging of myelin. *J. Neurosci. Meth.* 209 344–350. 10.1016/j.jneumeth.2012.06.015 22743799 PMC3429707

[B46] Natt O. Watanabe T. Boretius S. Radulovic J. Frahm J. Michaelis T. (2002). High-resolution 3D MRI of mouse brain reveals small cerebral structures in vivo. *J. Neurosci. Meth.* 120 203–209. 10.1016/s0165-0270(02)00211-x 12385770

[B47] Palermo F. Pieroni N. Sanna A. Parodi B. Venturi C. Provinciali G. (2022). Multilevel X-ray imaging approach to assess the sequential evolution of multi-organ damage in multiple sclerosis. *Commun. Phys.* 5:290.

[B48] Parlanti P. Cappello V. Brun F. Tromba G. Rigolio R. Tonazzini I. (2017). Size and specimen-dependent strategy for x-ray micro-ct and tem correlative analysis of nervous system samples. *Sci. Rep.* 7:2858. 10.1038/s41598-017-02998-1 28588216 PMC5460131

[B49] Paschke N. Neumann W. Uhrig T. Winkler M. Neumaier-Probst E. Fatar M. (2018). Influence of gadolinium-based contrast agents on tissue sodium quantification in sodium magnetic resonance imaging. *Invest. Radiol.* 53 555–562. 10.1097/RLI.0000000000000487 29863602

[B50] Perens J. Hecksher-Sorensen J. (2022). Digital brain maps and virtual neuroscience: An emerging role for light-sheet fluorescence microscopy in drug development. *Front. Neurosci.* 16:866884. 10.3389/fnins.2022.866884 35516798 PMC9067159

[B51] Pinto R. Matula J. Gomez-Lazaro M. Sousa M. Lobo A. Zikmund T. (2022). High-resolution micro-CT for 3D infarct characterization and segmentation in mice stroke models. *Sci. Rep.* 12:17471. 10.1038/s41598-022-21494-9 36261475 PMC9582034

[B52] Rawson S. Maksimcuka J. Withers P. Cartmell S. H. (2020). X-ray computed tomography in life sciences. *BMC Biol.* 18:21. 10.1186/s12915-020-0753-2 32103752 PMC7045626

[B53] Ribi W. Senden T. Sakellariou A. Limaye A. Zhang S. (2008). Imaging honey bee brain anatomy with micro-X-ray-computed tomography. *J Neurosci. Methods* 171 93–97. 10.1016/j.jneumeth.2008.02.010 18400304

[B54] Rodgers G. Kuo W. Schulz G. Scheel M. Migga A. Bikis C. (2021). Virtual histology of an entire mouse brain from formalin fixation to paraffin embedding. Part 1: Data acquisition, anatomical feature segmentation, tracking global volume and density changes. *J. Neurosci. Methods* 364:109354. 10.1016/j.jneumeth.2021.109354 34529981

[B55] Rodgers G. Tanner C. Schulz G. Migga A. Kuo W. Bikis C. (2022). Virtual histology of an entire mouse brain from formalin fixation to paraffin embedding. Part 2: Volumetric strain fields and local contrast changes. *J. Neurosci. Methods* 365:109385. 10.1016/j.jneumeth.2021.109385 34637810

[B56] Rodrigues P. Tostes K. Bosque B. de Godoy J. Amorim Neto D. Dias C. (2021). Illuminating the brain with X-Rays: Contributions and future perspectives of high-resolution microtomography to neuroscience. *Front. Neurosci.* 15:627994. 10.3389/fnins.2021.627994 33815039 PMC8010130

[B57] Rother L. Kraft N. Smith D. el Jundi B. Gill R. Pfeiffer K. (2021). A micro-CT-based standard brain atlas of the bumblebee. *Cell Tissue Res.* 386 29–45. 10.1007/s00441-021-03482-z 34181089 PMC8526489

[B58] Saito S. Murase K. (2012). Ex vivo imaging of mouse brain using micro-CT with non-ionic iodinated contrast agent: A comparison with myelin staining. *Br. J. Radiol.* 85 e973–e978. 10.1259/bjr/13040401 22674712 PMC3500820

[B59] Saito S. Murase K. (2013). Visualization of mouse spinal cord microscopic structures by use of ex vivo quantitative micro-CT images. *Radiol. Phys. Technol.* 6 7–13. 10.1007/s12194-012-0163-4 22729620

[B60] Schindelin J. Arganda-Carreras I. Frise E. Kaynig V. Longair M. Pietzsch T. (2012). Fiji: An open-source platform for biological-image analysis. *Nat. Methods* 9 676–682. 10.1038/nmeth.2019 22743772 PMC3855844

[B61] Schröder H. Moser N. Huggenberger S. (2020). *Neuroanatomy of the mouse: An introduction.* Berlin: Springer.

[B62] Siegel G. (2006). *Basic neurochemistry: Molecular, cellular and medical aspects.* Amsterdam: Academic Press.

[B63] Smith D. Bernhardt G. Raine N. Abel R. Sykes D. Ahmed F. (2016). Exploring miniature insect brains using micro-CT scanning techniques. *Sci. Rep.* 6:21768. 10.1038/srep21768 26908205 PMC4764865

[B64] Stewart R. Patwa A. Lusic H. Freedman J. Wathier M. Snyder B. (2017). Synthesis and preclinical characterization of a cationic iodinated imaging contrast agent (CA4+) and its use for quantitative computed tomography of ex vivo human hip cartilage. *J. Med. Chem.* 60 5543–5555. 10.1021/acs.jmedchem.7b00234 28616978 PMC6408935

[B65] Susaki E. Shimizu C. Kuno A. Tainaka K. Li X. Nishi K. (2020). Versatile whole-organ/body staining and imaging based on electrolyte-gel properties of biological tissues. *Nat. Commun.* 11:1982. 10.1038/s41467-020-15906-5 32341345 PMC7184626

[B66] Takeda T. Kunii T. Sirai R. Ohizumi T. Maruyama H. Hyodo K. (2013). Ethanol fixed brain imaging by phase-contrast X-ray technique. *J. Phys.* 425:022004.

[B67] Taranda J. Turcan S. (2021). 3D whole-brain imaging approaches to study brain tumors. *Cancers* 13:1897. 10.3390/cancers13081897 PMC8071100 33920839

[B68] Torkildsen O. Brunborg L. Myhr K. Bo L. (2008). The cuprizone model for demyelination. *Acta Neurol. Scand.* 117 72–76. 10.1111/j.1600-0404.2008.01036.x 18439226

[B69] Udagawa S. Miyara K. Takekata H. Takeuchi Y. Takemura A. (2019). Investigation on the validity of 3D micro-CT imaging in the fish brain. *J. Neurosci. Methods* 328:108416. 10.1016/j.jneumeth.2019.108416 31472188

[B70] Van Rompuy L. Parac-Vogt T. (2019). Interactions between polyoxometalates and biological systems: From drug design to artificial enzymes. *Curr. Opin. Biotech.* 58 92–99. 10.1016/j.copbio.2018.11.013 30529815

[B71] Vandebroek L. De Zitter E. Ly H. Conic D. Mihaylov T. Sap A. (2018). Protein-assisted formation and stabilization of catalytically active polyoxometalate species. *Chem. Eur. J.* 24 10099–10108. 10.1002/chem.201802052 29797738

[B72] Velegol D. Garg A. Guha R. Kar A. Kumar M. (2016). Origins of concentration gradients for diffusiophoresis. *Soft Matter* 12 4686–4703. 27174044 10.1039/c6sm00052e

[B73] Veuthey T. Herrera G. Dodero V. (2014). Dyes and stains: From molecular structure to histological application. *Front. Biosci.* 19 91–112. 10.2741/4197 24389174

[B74] Vickerton P. Jarvis J. Jeffery N. (2013). Concentration-dependent specimen shrinkage in iodine-enhanced microCT. *J. Anat.* 223 185–193. 23721431 10.1111/joa.12068 PMC3724211

[B75] Wang Y. Pan Y. Li H. (2020). What is brain health and why is it important? *BMJ Brit. Med. J.* 371:m3683. 10.1136/bmj.m3683 PMC7555053 33037002

[B76] White G. Brown C. (2015). Variation in brain morphology of intertidal gobies: A comparison of methodologies used to quantitatively assess brain volumes in fish. *Brain Behav. Evol.* 85 245–256. 10.1159/000398781 26183604

[B77] Wong M. Spring S. Henkelman R. (2013). Structural stabilization of tissue for embryo phenotyping using micro-CT with iodine staining. *PLoS One* 8:e84321. 10.1371/journal.pone.0084321 24386367 PMC3875560

[B78] Xie M. Tobin J. Budde M. Chen C. Trinkaus K. Cross A. (2010). Rostrocaudal analysis of corpus callosum demyelination and axon damage across disease stages refines diffusion tensor imaging correlations with pathological features. *J. Neuropath Exp. Neur.* 69 704–716. 10.1097/NEN.0b013e3181e3de90 20535036 PMC2901930

[B79] Zikmund T. Novotna M. Kavkova M. Tesarova M. Kaucka M. Szarowska B. (2018). High-contrast differentiation resolution 3D imaging of rodent brain by X-ray computed microtomography. *J. Instrum.* 13 1–12.

[B80] Zirngibl M. Assinck P. Sizov A. Caprariello A. Plemel J. (2022). Oligodendrocyte death and myelin loss in the cuprizone model: An updated overview of the intrinsic and extrinsic causes of cuprizone demyelination. *Mol. Neurodegener.* 17:34. 10.1186/s13024-022-00538-8 35526004 PMC9077942

