# Supplementary material for: Revealing the three-dimensional murine brain microstructure by contrast-enhanced computed tomography
Source: Front Neurosci. 2023 Mar 23;17:1141615. doi: 10.3389/fnins.2023.1141615 (PMC10076597; doi:10.3389/fnins.2023.1141615)
Supplement: Supplementary file 1 [file Data_Sheet_1.docx]

Supplementary Material

Revealing the three-dimensional murine brain microstructure by contrast-enhanced computed tomography

Tim Balcaen^1,2,3^, Catherine Piens^2^, Ariane Mwema^4,5^, Matthieu Chourrout^6^, Laurens Vandebroek^7^, Anne Des Rieux^4^, Fabien Chauveau^6^, Wim M. De Borggraeve^1^, Delia Hoffmann^3,8,9,†^ & Greet Kerckhofs^2,3,10,†,*^

^1^MolDesignS, Sustainable Chemistry for Metals and Molecules, Department of Chemistry, KU Leuven, Leuven, Belgium

^2^ContrasT Team, Institute of Mechanics, Materials and Civil Engineering, Mechatronic, Electrical Energy and Dynamic Systems, UCLouvain, Louvain-la-Neuve, Belgium

^3^Pole of Morphology, Institute of Experimental and Clinical Research, UCLouvain, Brussels, Belgium

^4^Advanced Drug Delivery and Biomaterials, UCLouvain, Brussels, Belgium

^5^Bioanalysis and pharmacology of bioactive lipids, UCLouvain, Brussels, Belgium

^6^ Université Claude Bernard Lyon 1, Centre National de la Recherche Scientifique, Institut National de la Santé et de la Recherche Médicale, Centre de Recherche en Neurosciences de Lyon U1028 UMR5292, BIORAN, F-69500, Bron, France

^7^Lab of Biomolecular Modelling and Design (LBMD), Biochemistry, Molecular and Structural Biology, Department of Chemistry KU Leuven, Leuven, Belgium

^8^Prometheus, Division of Skeletal Tissue Engineering, KU Leuven, Leuven, Belgium

^9^Skeletal Biology and Engineering Research Center, KU Leuven, Leuven, Belgium

^10^Department Materials Engineering, KU Leuven, Leuven, Belgium

^†^These authors contributed equally to this work and share last authorship

*** Correspondence:**Greet Kerckhofs
greet.kerckhofs@uclouvain.be

# Supplementary Figures and Tables

## Supplementary Figures

**

Supplementary figure 1: Correlation functions that were measured and used to determine the particle size distributions using dynamic light scattering.


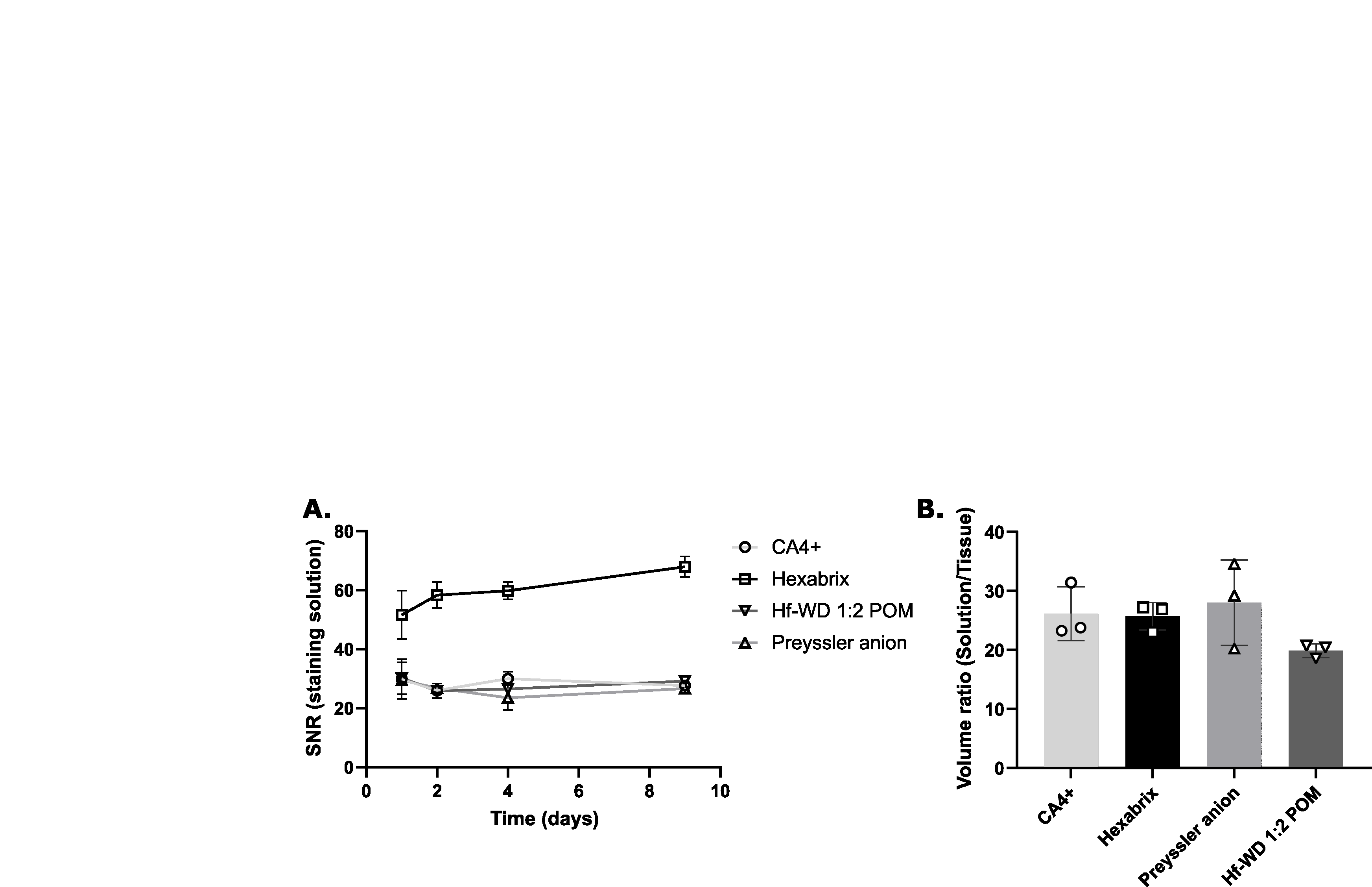


**Supplementary figure 2**: A. Graph illustrating the change of SNR of the staining solution over time per CESA. B. Bar plot of the volume ratio (Solution/Tissue).

Supplementary figure 3: Statistical comparison between weight of each mouse during cuprizone treatment (9/06/2022 – 20/07/2022. Full model Two-way ANOVA (with Geisser-Greenhouse correction) followed by Bonferroni multiple comparisons test (week 6: p-value = 0.0426, 95%CI [0.152, 8.641]; week 7: p-value = 0.045, 95%CI [0.1140-9.376]).

## Supplementary tables

Supplementary table 1: Overview of previously reported CECT studies on nervous tissue with respective animals and applied CESAs.

| **Animal** | **CESA Class** | **CESA** | **References** |
| --- | --- | --- | --- |
| Bumblebee | Inorganic | Uranyl acetate | (1, 2) |
|  |  | Phosphotungstic acid |  |
|  |  | Iodine in Ethanol |  |
| Fish | Inorganic | Osmium tetroxide | (3) |
|  | Organic | Iopamiron® | (4) |
| Fruit fly larvae | Inorganic | Silver nitrate | (5) |
| Honey bee | Inorganic | Osmium tetroxide | (6) |
| Human | Inorganic | Lugol’s iodine | (7, 8) |
| Mouse | Inorganic | Uranyl acetate | (9-18) |
|  |  | Osmium tetroxide |  |
|  |  | Lugol’s iodine |  |
|  |  | Iodine in Ethanol  Iodine in Methanol |  |
|  |  |  |  |
|  | Organic | Hypaque®-76 | (19-23) |
|  |  | Iopamiron® |  |
|  |  | Visipaque®  µAngiofil® |  |
|  |  |  |  |
| Rabbit | Organic | Hypaque®-76 | (19) |
| Rat | Inorganic | Iodine in Methanol  Iodine in Ethanol | (12, 24-27) |
|  |  | Osmium tetroxide |  |
|  |  | Uranyl acetate  Phosphotungstic acid |  |
| Sheep | Inorganic | Potassium dichromate | (28) |

Supplementary table 2: A. Weight of each mouse during cuprizone treatment (9/06/2022 – 20/07/2022). B. Food intake of mice per cage during cuprizone treatment (9/06/2022 – 20/07/2022).

|  |  | **Weight (g)** | | | | | | |
| --- | --- | --- | --- | --- | --- | --- | --- | --- |
|  | **A.** | Week 1 | Week 2 | Week 3 | Week 4 | Week 5 | Week 6 | Week 7 |
| Cage 1 Control | Mouse 1 | 22.37 | 23.21 | 24.87 | 26.31 | 27.7 | 28.71 | 28.68 |
|  | Mouse 2 | 23.95 | 24.61 | 25.56 | 27.32 | 28.11 | 29.62 | 28.9 |
|  | Mouse 3 | 20.59 | 18.55 | 21.37 | 22.94 | 23.32 | 25.01 | 25.18 |
|  | Mouse 4 | 23.91 | 24.34 | 26.55 | 27.4 | 28.73 | 30.38 | 30.47 |
|  | Mouse 5 | 22.61 | 22.77 | 23.53 | 24.11 | 24.4 | 25.92 | 25.6 |
|  | Mouse 6 | 20.59 | 18.55 | 21.37 | 22.94 | 23.32 | 25.01 | 25.18 |
|  | Mean | 22.34 | 22.01 | 23.88 | 25.17 | 25.93 | 27.44 | 27.34 |
|  | Std. dev. | 1.50 | 2.76 | 2.18 | 2.10 | 2.52 | 2.41 | 2.30 |
| Cage 2 Cuprizone | Mouse 1 | 22.93 | 20.61 | 20.74 | 21.83 | 21.55 | 22.66 | 22 |
|  | Mouse 2 | 23.75 | 22.36 | 22.33 | 22.04 | 22.14 | 23.43 | 23.18 |
|  | Mean | 23.34 | 21.49 | 21.54 | 21.94 | 21.85 | 23.05 | 22.59 |
|  | Std. dev. | 0.58 | 1.24 | 1.12 | 0.15 | 0.42 | 0.54 | 0.83 |

|  |  | **Food per cage (g)** | | | | | | |
| --- | --- | --- | --- | --- | --- | --- | --- | --- |
|  | **B.** | Week 1 | Week 2 | Week 3 | Week 4 | Week 5 | Week 6 | Week 7 |
| Cage 1 Control | Food added | 211.53 | 210.4 | 209.92 | 210.57 | 210.58 | 210.62 | Sacrifice |
|  | Food left | 92.95 | 86.09 | 83.5 | 85.03 | 72.26 | 108.42 |  |
|  | Food eaten | 118.58 | 124.31 | 126.42 | 125.54 | 138.32 | 102.2 |  |
| Cage 2 Cuprizone | Food added | 212.96 | 211.3 | 209.7 | 209.77 | 210.56 | 210.83 |  |
|  | Food left | 52.68 | 123.37 | 112.97 | 121.4 | 107.63 | 132.04 |  |
|  | Food eaten | 160.28 | 87.93 | 96.73 | 88.37 | 102.93 | 78.79 |  |

Supplementary table 3: Overview of cylinder correlation algorithm parameters for annotation of white matter fiber bundles to generate thickness distributions.

| **Cylinder correlation** | |
| --- | --- |
| **Input parameter** | **Value** |
| Cylinder length (µm) | 150 |
| Angular sampling | 5 |
| Mask cylinder radius (µm) | 20 |
| Outer cylinder radius (µm) | 10 |
| Inner cylinder radius (µm) | 0 |
| Contrast | Dark on Bright |
| **Trace correlation lines** | |
| **Input parameter** | **Value** |
| Minimum seed correlation | 120 |
| Minimum continuation quality | 100 |
| Direction coefficient | 0.3 |
| Minimum distance (µm) | 20 |
| Minimum length (µm) | 150 |
| Search cone length (µm) | 150 |
| Search cone angle (°) | 37 |
| Minimum step size (%) | 10 |

Supplementary table 4: Computed physicochemical properties (ACD/Labs^®^) of a myelin-specific fluorescent dye, CA4+, Hexabrix and previously used organic, iodinated CESAs in CECT. *cLogD computed at pH = 7.4.

| **Molecule** | **cLogP** | **cLogD*** | **Net charge** |
| --- | --- | --- | --- |
| FluoroMyelin Dye(29) | -2.27 | -2.28 | +2 |
| CA4+ | -3.61 | -8.3 | +4 |
| Hexabrix | 1.62 | -1.52 | -1 |
| Iopamiron(4, 20) | -2.31 | -2.31 | 0 |
| Hypaque-76(19) | 2.14 | -1.00 | -1 |
| Visipaque(22) | -3.73 | -3.73 | 0 |

# **Bibliograph**y

1. Rother L, Kraft N, Smith DB, el Jundi B, Gill RJ, Pfeiffer K. A micro-CT-based standard brain atlas of the bumblebee. Cell Tissue Res. 2021;386(1):29-45.

2. Smith DB, Bernhardt G, Raine NE, Abel RL, Sykes D, Ahmed F, et al. Exploring miniature insect brains using micro-CT scanning techniques. Sci Rep. 2016;6:21768.

3. White GE, Brown C. Variation in Brain Morphology of Intertidal Gobies: A Comparison of Methodologies Used to Quantitatively Assess Brain Volumes in Fish. Brain Behav Evol. 2015;85(4):245-56.

4. Udagawa S, Miyara K, Takekata H, Takeuchi Y, Takemura A. Investigation on the validity of 3D micro-CT imaging in the fish brain. J Neurosci Methods. 2019;328:108416.

5. Mizutani R, Takeuchi A, Hara T, Uesugi K, Suzuki Y. Computed tomography imaging of the neuronal structure of Drosophila brain. J Synchrotron Radiat. 2007;14(Pt 3):282-7.

6. Ribi W, Senden TJ, Sakellariou A, Limaye A, Zhang S. Imaging honey bee brain anatomy with micro-X-ray-computed tomography. J Neurosci Methods. 2008;171(1):93-7.

7. Lombardi S, Scola E, Ippolito D, Zambelli V, Botta G, Cuttin S, et al. Micro-computed tomography: a new diagnostic tool in postmortem assessment of brain anatomy in small fetuses. Neuroradiology. 2019;61(7):737-46.

8. Feldman KM, O'Keefe YA, Gignac PM, O'Brien HD. Highest resolution microCT scan of the human brainstem reveals putative anatomical basis for infrequency of medial medullary syndrome. Neuroimage Clin. 2022;36:103272.

9. Prajapati SI, Kilcoyne A, Samano AK, Green DP, McCarthy SD, Blackman BA, et al. MicroCT-based virtual histology evaluation of preclinical medulloblastoma. Mol Imaging Biol. 2011;13(3):493-9.

10. Anderson R, Maga AM. A Novel Procedure for Rapid Imaging of Adult Mouse Brains with MicroCT Using Iodine-Based Contrast. PLoS One. 2015;10(11):e0142974.

11. Choi JP, Yang X, Foley M, Wang X, Zheng X. Induction and Micro-CT Imaging of Cerebral Cavernous Malformations in Mouse Model. J Vis Exp. 2017(127).

12. Parlanti P, Cappello V, Brun F, Tromba G, Rigolio R, Tonazzini I, et al. Size and specimen-dependent strategy for x-ray micro-ct and tem correlative analysis of nervous system samples. Sci Rep. 2017;7(1):2858.

13. Llambrich S, Wouters J, Himmelreich U, Dierssen M, Sharpe J, Gsell W, et al. ViceCT and whiceCT for simultaneous high-resolution visualization of craniofacial, brain and ventricular anatomy from micro-computed tomography. Sci Rep. 2020;10(1):18772.

14. Buytaert J, Goyens J, De Greef D, Aerts P, Dirckx J. Volume shrinkage of bone, brain and muscle tissue in sample preparation for micro-CT and light sheet fluorescence microscopy (LSFM). Microsc Microanal. 2014;20(4):1208-17.

15. Heimel P, Swiadek NV, Slezak P, Kerbl M, Schneider C, Nurnberger S, et al. Iodine-Enhanced Micro-CT Imaging of Soft Tissue on the Example of Peripheral Nerve Regeneration. Contrast Media Mol Imaging. 2019;2019:7483745.

16. Zikmund T, Novotna M, Kavkova M, Tesarova M, Kaucka M, Szarowska B, et al. High-contrast differentiation resolution 3D imaging of rodent brain by X-ray computed microtomography. J Instrum. 2018;13.

17. Pinto R, Matula J, Gomez-Lazaro M, Sousa M, Zikmund T, Kaiser J, et al. High resolution micro-CT imaging in mice stroke models: from 3D detailed infarct characterization to automatic area segmentation. bioRxiv. 2022:2022.02.02.478782.

18. Pinto R, Matula J, Gomez-Lazaro M, Sousa M, Lobo A, Zikmund T, et al. High-resolution micro-CT for 3D infarct characterization and segmentation in mice stroke models. Sci Rep. 2022;12(1):17471.

19. de Crespigny A, Bou-Reslan H, Nishimura MC, Phillips H, Carano RA, D'Arceuil HE. 3D micro-CT imaging of the postmortem brain. J Neurosci Methods. 2008;171(2):207-13.

20. Saito S, Murase K. Ex vivo imaging of mouse brain using micro-CT with non-ionic iodinated contrast agent: a comparison with myelin staining. Br J Radiol. 2012;85(1019):e973-8.

21. Saito S, Murase K. Visualization of mouse spinal cord microscopic structures by use of ex vivo quantitative micro-CT images. Radiol Phys Technol. 2013;6(1):7-13.

22. Bukreeva I, Asadchikov V, Buzmakov A, Chukalina M, Ingacheva A, Korolev NA, et al. High resolution 3D visualization of the spinal cord in a post-mortem murine model. Biomed Opt Express. 2020;11(4):2235-53.

23. Hlushchuk R, Haberthur D, Soukup P, Barre SF, Khoma OZ, Schittny J, et al. Innovative high-resolution microCT imaging of animal brain vasculature. Brain Struct Funct. 2020;225(9):2885-95.

24. Masis J, Mankus D, Wolff SBE, Guitchounts G, Joesch M, Cox DD. A Micro-CT-based Method for Characterizing Lesions and Locating Electrodes in Small Animal Brains. J Vis Exp. 2018(141).

25. Masis J, Mankus D, Wolff SBE, Guitchounts G, Joesch M, Cox DD. A micro-CT-based method for quantitative brain lesion characterization and electrode localization. Sci Rep. 2018;8(1):5184.

26. Kavkova M, Zikmund T, Kala A, Salplachta J, Proskauer Pena SL, Kaiser J, et al. Contrast enhanced X-ray computed tomography imaging of amyloid plaques in Alzheimer disease rat model on lab based micro CT system. Sci Rep. 2021;11(1):5999.

27. Chen KC, Arad A, Song ZM, Croaker D. High-definition neural visualization of rodent brain using micro-CT scanning and non-local-means processing. Bmc Med Imaging. 2018;18.

28. Herrera M, Notario B, Barrio MC, Metscher BD, Murillo Gonzalez J. X-ray micro-computed tomography of postmortem brain tissue using potassium dichromate as a contrast agent. Arch Ital Biol. 2018;156(1-2):48-53.

29. Kilgore, J. J. (2006), Lipophilic dyes and their applications for detection of myelin. US 2006/0073541 A1. United States Patent Application Publication.
